# Transcriptome Analysis of a New Peanut Seed Coat Mutant for the Physiological Regulatory Mechanism Involved in Seed Coat Cracking and Pigmentation

**DOI:** 10.3389/fpls.2016.01491

**Published:** 2016-10-14

**Authors:** Liyun Wan, Bei Li, Manish K. Pandey, Yanshan Wu, Yong Lei, Liying Yan, Xiaofeng Dai, Huifang Jiang, Juncheng Zhang, Guo Wei, Rajeev K. Varshney, Boshou Liao

**Affiliations:** ^1^Key Laboratory of Biology and Genetic Improvement of Oil Crops, Ministry of Agriculture, Oil Crops Research Institute of Chinese Academy of Agricultural SciencesWuhan, China; ^2^Center of Excellence in Genomics, International Crops Research Institute for the Semi-Arid TropicsHyderabad, India; ^3^Institute of Food Science and Technology of Chinese Academy of Agricultural SciencesBeijing, China; ^4^School of Plant Biology and Institute of Agriculture, The University of Western AustraliaCrawley, WA, Australia

**Keywords:** peanut (*Arachis hypogaea*), seed-coat cracking, pigmentation, RNA-seq, flavonoid pathway

## Abstract

Seed-coat cracking and undesirable color of seed coat highly affects external appearance and commercial value of peanuts (*Arachis hypogaea* L.). With an objective to find genetic solution to the above problems, a peanut mutant with cracking and brown colored seed coat (testa) was identified from an EMS treated mutant population and designated as “peanut seed coat crack and brown color mutant line (*pscb*).” The seed coat weight of the mutant was almost twice of the wild type, and the germination time was significantly shorter than wild type. Further, the mutant had lower level of lignin, anthocyanin, proanthocyanidin content, and highly increased level of melanin content as compared to wild type. Using RNA-Seq, we examined the seed coat transcriptome in three stages of seed development in the wild type and the *pscb* mutant. The RNA-Seq analysis revealed presence of highly differentially expressed phenylpropanoid and flavonoid pathway genes in all the three seed development stages, especially at 40 days after flowering (DAF40). Also, the expression of polyphenol oxidases and peroxidase were found to be activated significantly especially in the late seed developmental stage. The genome-wide comparative study of the expression profiles revealed 62 differentially expressed genes common across all the three stages. By analyzing the expression patterns and the sequences of the common differentially expressed genes of the three stages, three candidate genes namely *c36498_g1 (CCoAOMT1), c40902_g2 (kinesin)*, and *c33560_g1 (MYB3)* were identified responsible for seed-coat cracking and brown color phenotype. Therefore, this study not only provided candidate genes but also provided greater insights and molecular genetic control of peanut seed-coat cracking and color variation. The information generated in this study will facilitate further identification of causal gene and diagnostic markers for breeding improved peanut varieties with smooth and desirable seed coat color.

## Introduction

A typical peanut (*Arachis hypogaea* L.) seed has three parts i.e., seed coat (also known as testa), embryo and endosperm. Seed coat is the outer protective layer of seed and one of its major role is to provide protection to embryo and endosperm from external factors such as infection of insects, bacteria, fungi and virus, mechanical injuries, and even desiccation of the seed. In legumes including peanut, the seed coat, and endosperm develop first, followed by the embryo (Weber et al., 2005). Rapid cotyledon growth sometimes may not adequately match the expansion of the seed coat leading to formation of cracks in seed coat (Agarwal and Menon, 1974). In other words, the seed-coat cracking (SC) results from the separation of epidermal (palisade cells) and hypodermal tissues leading to exposure of the underlying parenchyma tissues (Wolf and Baker, 1972). The most adverse effect of SC is that seeds become more vulnerable to storage problems and field microorganisms, leading to seed rotting or pre- and post-emergence damping under high humid conditions. Although the reason for the physical separation of palisade and hypodermal cells is not well-known, genetic and environmental factors have been implicated for SC in other crops such as soybean (Stewart and Wentz, 1930; Woodworth and Williams, 1938; Liu, 1949; Schlub and Schmitthenner, 1978; Duke et al., 1983, 1986) and watermelon (Hafez et al., 1981). The seed coat cracks in soybean were linked to physiological and ultimate structure of the cell wall (Kour et al., 2014).

Lignin is a complex and heterogeneous polymer that constitutes one of the major components of the secondary wall of xylem cells and fibers (Mellerowicz et al., 2001). Lignification confers not only the mechanical support and optimizes transport of water and solutes along vascular system but also protects against pathogens (Boerjan et al., 2003). Lignin is the second most abundant biological product in nature, and is formed by oxidative polymerization of three main constituents, namely monolignols p-coumaryl, coniferyl, and sinapyl alcohols through the phenylpropanoid pathway. Once incorporated in the lignin polymer, these precursors are known as p-hydroxyphenyl (H), guaiacyl (G), and syringyl (S) subunits, respectively (Anterola and Lewis, 2002; Boerjan et al., 2003). Lignins together with anthocyanins, flavonols and proanthocyanidins constitute the main group of plant phenylpropanoids (Fornalé et al., 2010).

The seed coat color varies in different species and genotypes and even at different seed developmental stages. Flavonoids are the major secondary metabolites influencing seed coat color in plants and represent a highly diverse group of plant aromatic secondary metabolites. The major forms of flavonoids include anthocyanins (red to purple pigments), flavonols (colorless to pale yellow pigments), and proanthocyanidins (PAs), also known as condensed tannins (colorless pigments that brown with oxidation). These flavonoids are present in varied proportions and quantity in different plant species, organs, developmental stages and environmental conditions. The PAs, oligomers of flavan-3-ol units, have received particular attention due to their abundance in seed coats (Dixon et al., 2005). The mechanism of seed coat pigmentation was well-studied in model plants such as *Arabidopsis* wherein the flavonols and proanthocyanidins derivatives were found responsible for the pigmentation pattern of seeds in addition to their involvement in a wide range of biological functions (Shirley et al., 1995). In the mature testa, flavonoids were detected in both the endothelium and the three crushed parenchymal layers just above the endothelium (Debeaujon et al., 2000). PAs have been shown to accumulate exclusively in the endothelium layer (Devic et al., 1999).

In recent years, several efforts were made for elucidating the flavonoid biosynthetic pathway from the molecular genetics point of view (Winkel-Shirley, 2001; Tanaka et al., 2008). Mutants affecting flavonoid synthesis were isolated in a variety of plant species based on alterations in flower and seed pigmentation. Recent studies conducted in *Arabidopsis* helped in developing some understanding on regulation and subcellular organization of the flavonoid pathway (Winkel-Shirley, 2001). The same study also indicated that the genetic loci for both structural and regulatory genes were scattered across the *Arabidopsis* genome and were identified largely on the basis of mutations that abolish or reduce pigmentation in the seed coat. The major functional and regulatory genes in flavonoid metabolism include *PAL, C4H, 4CL, CHS(TT4), CHI(TT5), F3H(TT6), F3*′*H(TT7), DRF(TT3), ANS(LODX*\*TT18), LAR/LCR, BAN(ANR), TT12, TT19(GST), TT10, FLS*, and *AHA10* etc. (Chapple et al., 1994; Winkel-Shirley, 2001; Abrahams et al., 2003). Regulatory proteins controlling flavonoid biosynthesis were also characterized e.g., MYB–bHLH–WDR (MBW) complex was found to be involved in biosynthesis of PAs and anthocyanins (Baudry et al., 2006; Lepiniec et al., 2006) and the R2R3-MYBs PRODUCTION OF FLAVONOL GLYCOSIDE (PFG1/MYB12, PFG2/MYB11, and PFG3/MYB111) positively regulated flavonol biosynthesis in root and the aerial parts (Dubos et al., 2010; Stracke et al., 2010a,b), whereas single repeat small MYBs CAPRICE (CPC) or MYBL2 was found to be involved in negatively regulating anthocyanin synthesis (Dubos et al., 2008; Zhu et al., 2009).

In peanut, previous studies mainly focused on identifying the antioxidant of seed coat and the extraction pigments (Wang et al., 2007; Ballard et al., 2009; Zhang et al., 2013; de Camargo et al., 2014; Ma et al., 2014). These studies showed that peanut seed coat with different colors were composed of different pigment composition. However, none of the above mentioned studies provided any information on peanut seed-coat cracking and pigmentation mechanism. In this study, we first identified a spontaneous seed coat-cracking and seed color mutant from Zhonghua16, designated as “*pscb*,” and then employed RNA-seq approach to develop better understanding of the mechanism of seed coat-cracking and brown color development in seed coat of peanut.

## Materials and methods

### Plant materials and RNA isolation

The seed coat crack and brown testa mutant *pscb* was isolated from an ethyl methanesulfonate (EMS)-mutated population originated from an improved peanut cultivar, Zhonghua 16, with high yield and high oil content. All plants were planted in the experimental farm at the Oil Crops Research Institute (OCRI) in Wuhan.

The wild type (WT) and *pscb* mutant (M6 generation) were planted in the same field (Wuhan, China). Seed coat samples were taken at 20, 40, and 60 days after flowering (DAF) from 10 different plants in 2014. Twelve representative seeds were sampled from each seedling at each developmental stage of both the wild type and the mutant. Three biological replicates were designed. The testa separated from the sample seeds was sliced. The sliced WT and the *pscb* mutant testa samples were then frozen rapidly in liquid nitrogen and kept at −80°C, and were later used for extracting the RNA using the Tiangen RNA extraction kit (category number DP432).

### Seed water uptake and germination assays

Seeds used for permeability study were harvested in 2014. For the WT and *pscb* materials, 30 seeds were tested with three replicates. The seeds were weighed, immersed in tap water for each specific time, removed from the water, blotted with cellulose tissue, weighed again, and kept again into the water. Seeds were weighed at the intervals of 30-min and 60-min during the first 8 h; at 60-min intervals during the last 6 h; and a final measurement at 24 h. The rate of water uptake was calculated by expressing it as weight increase (g) per gram seed weight (initial).

For the seed germination test, seeds were incubated in petri dishes (9 cm diameter) over two layers of medium-speed qualitative filter papers. A total of 20 seeds were placed in each petri dish and added 12 ml of sterile water. Complete experiment was performed in three replications. The seeds were incubated in a 25°C incubator with darkness. Germination was determined based on the radicle breaking through the seed coat. The germination percentage was calculated and recorded at different time points.

### Tissue preparation and light microscopy observations

Peanut seeds were harvested at 20, 30, 40, 50, and 60 DAF and immediately were fixed for 24 h at 4°C in a fixation solution containing 5% acetic acid, 5% formaldehyde, and 50% ethanol. Following fixation, seeds were dehydrated at 60 min intervals through a 20% step-graded series of ethanol-water mixtures, and ended at 100% ethanol. Then, the seeds were processed at 60 min intervals through a 30% step-graded series of ethanol-TBA (tert-butyl alcohol) mixtures, and ended at 100% TBA. Seeds were subsequently infiltrated over a 24 h period with saturated paraffin-TBA mixtures, and then embedded for 48 h period in paraffin. Blocks were completely polymerized at 4°C. Semi-thin (5–8 μm thick) sections were cut with a microtome blade KD-P (Zhejiang Jinhua Kedi Instrumental Equipment CO., LTD, China) and viewed under a stereo microscope (SZX12, Olympus, Japan). Sections were stained with TBO and observed with a Nikon ECLIPSE TI-SR microscope (Nikon Instruments, Japan).

### Quantification of lignin, anthocyanin, proanthocyanidin, and phytomelanin content

The lignin content was analyzed following Kirk and Obst (1988) and Hoebler et al. (1989), and the extraction of anthocyanins was performed as per Pang et al. (2009). For PA analysis, 0.5–0.75 g of ground samples were extracted using extraction solution containing 5 mL of 70% acetone/0.5% acetic acid. The samples were vortexed and then sonicated at room temperature for 1 h. Following centrifugation at 2500 g for 10 min, the residues were re-extracted twice following the same above mentioned procedure. The pooled supernatants were then extracted three times using chloroform, once with hexane. The supernatants (containing soluble PAs) and residues (containing insoluble PAs) from each sample were freeze dried separately and were then suspended in extraction solution. Total soluble PA content was determined using Spectrophotometer after reaction with DMACA reagent (0.2% [w/v] DMACA in methanol-3 N HCl) at 640 nm, with (+)-catechin as standard.

For quantification of insoluble PAs, 2 mL of butanol-HCl (95:5, v/v) was added to the dried residues and the mixtures were sonicated at room temperature for 1 h, followed by centrifugation at 2500 g for 10 min. The absorption of the supernatants was measured at 550 nm. The samples were then boiled for 1 h and cooled to room temperature, and were measured again. The values were recorded by subtracting the first value from the second. Absorbance values were converted into PA equivalents using a standard curve generated with procyanidin B1 (Indofine). The extraction and characterization of phytomelanins of mature seeds were described in detail by Park et al. (2007). The phytomelanin pigments were extracted from 1 g seeds in 5 mL of 0.5 M NaOH for 1 h. The extracts were purified, and were diluted 20 times with 0.5 M NaOH, and the dilutions were subjected to absorbance measurement at 280 nm using an Ultrospec EPOCH (BioTek, China).

### RNA-Seq, data processing, and gene annotation

According to the mutant seed coat color and crack phenotype, the WT and *pscb* mutant seed coat were harvested at DAF20, DAF40, and DAF60 followed by RNA sequencing using Illumina HiSeqTM2500 platform at the Novogene company (Beijing) in 2014. Briefly, 3 μg of the total RNA of each sample was used to enrich the mRNA and to construct cDNA libraries. High quality reads (clean reads) were obtained by removing low-quality reads with ambiguous nucleotides, and cutting the adaptor sequences. Transcripts were assembled using Trinity (Grabherr et al., 2011) while gene expression levels were calculated using RPKM (reads per kb per million reads) method of RSEM (Li and Dewey, 2011). Gene function was annotated based on multiple databases namely Nr (NCBI non-redundant protein sequences), Nt (NCBI non-redundant nucleotide sequences), Pfam (Protein family), KOG/COG (Clusters of Orthologous Groups of proteins), Swiss-Prot (A manually annotated and reviewed protein sequence database), KO (KEGG Ortholog database), and GO (Gene Ontology). The GO enrichment analysis of the differentially expressed genes (DEGs) was implemented by the GOseq R packages based Wallenius non-central hyper-geometric distribution (Young et al., 2010), which can adjust for gene length bias in DEGs. KOBAS (Mao et al., 2005) was used to perform KEGG pathway enrichment for the differential expression genes. Picard–tools (v1.41) and samtools (v0.1.18) were used to sort, remove duplicated reads and merge the bam alignment results of each sample.

### qRT-PCR analysis

The reverse transcriptions were performed using an Invitrogen SuperScript Reagent Kit. The primer was designed using the Oligo6 software. For RT-PCR, the SYBR® Premix ExTaq™ (TAKARA) was used on a Bio-Rad IQ5 real-time PCR detection system (Bio-Rad, Hercules, CA). Gene expression was analyzed for samples at 20, 40, and 60 DAF of WT and mutant. All reactions for each gene were performed in triplicate. The relative expression level of each gene among samples was calculated using the 2^−Δ*ΔCt*^ method with normalization to the internal reference actin gene. The parameters of thermal cycle were 95°C for 30 s, followed by 40 cycles of 95°C for 10 s and 50–56°C for 25 s at a volume of 20 μl.

## Results

### Phenotypic variation between seed coat of wild and mutant genotypes

**Figure 1:**
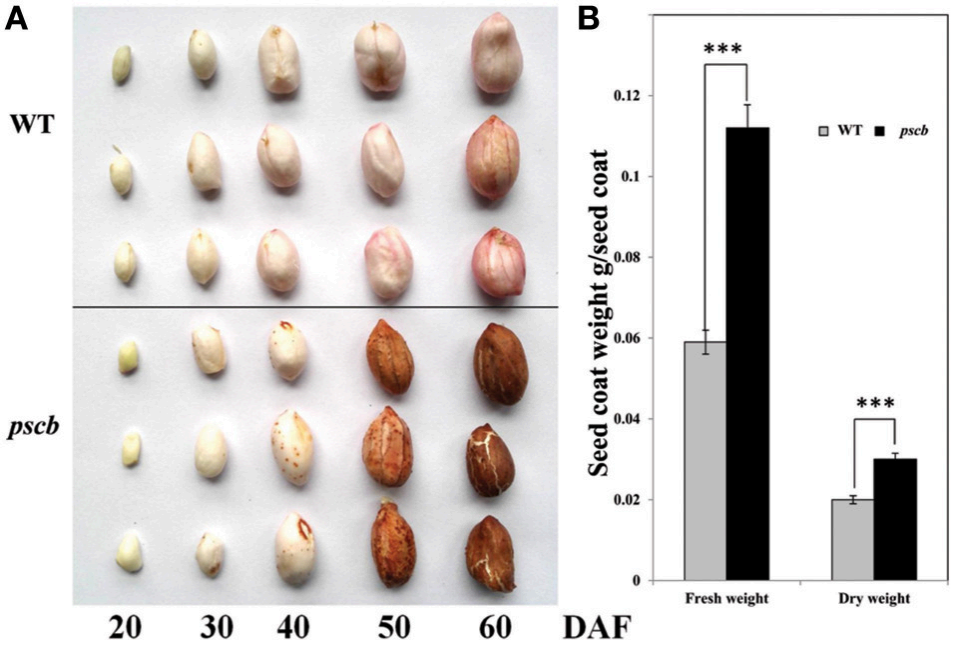
**Phenotypic characterization of peanut seed coat development in ***pscb*** mutant and WT. (A)** Phenotypic characterization of peanut seed coat development in *pscb* mutant and WT. **(B)** Fresh and dry weight of matured seed coat of *pscb* mutant and WT. ^***^Means significant differences.

A peanut mutant with cracked and brown color seed coat named *pscb* was identified from a mutant population treated with 1.0% EMS in the background of an elite peanut cultivar Zhonghua16 (WT). In M3 generation, the ratio of *pscb* mutant and the normal plants was 1:3 (69 *pscb* and 206 normal). Although there was no difference in the seed coat of WT and the *pscb* mutant at the early stage, however, few tiny brown points appeared in the seed coat of the *pscb* mutant at the stage of DAF40 when the seed coat of WT turned pink gradually. Interestingly, the seed coat of *pscb* turned totally brown while the WT were still in pink at DAF60. It was observed that the seed coat developed cracks when plants reached their physiological maturity and seeds were fully developed. At stage DAF60, seed coat cracks became more evident and wide (Figure 1). It is important to mention that cracks only appeared in the outer layer of the seed coat and not in the inner integument (Figure 1). The seed coat cracks in mutant appeared in all the growing conditions (3 years in Wuhan and 3 years in Zhanjiang) in varied intensities i.e., from a minute or invisible crack to several wide cracks. We observed that the mature seed coat of *pscb* mutant was much thicker than the WT. In addition, the seed coat of *pscb* mutant had 190% higher fresh weight and 150% higher dry weight than that of WT.

**Figure 2:**
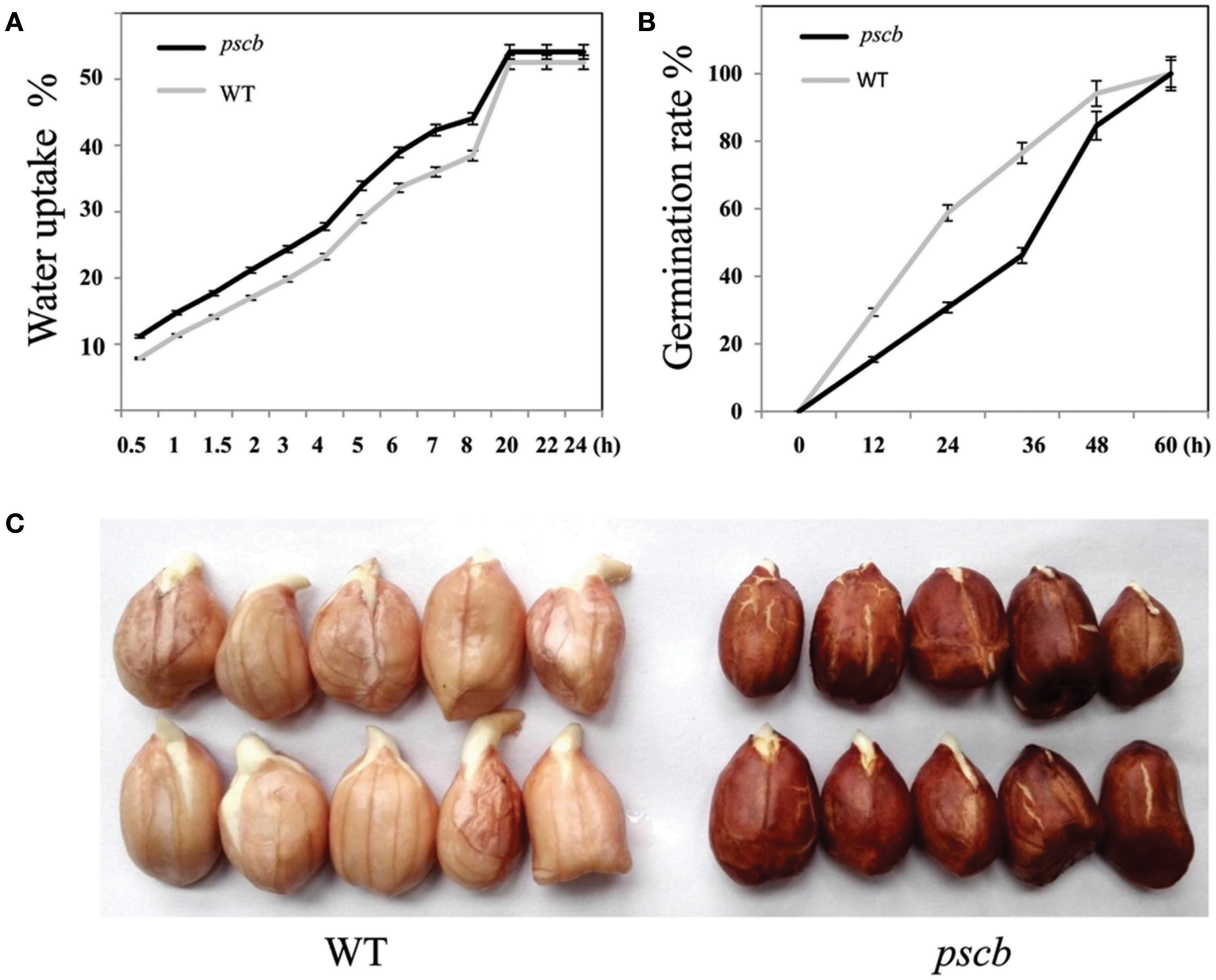
**Water uptake efficiency and germination in ***pscb*** mutant and WT. (A)** Water uptake efficiency of *pscb* mutant and WT. **(B)** Germination time of *pscb* mutant and WT. **(C)** The radicals elongated faster in WT when compared with *pscb* mutant.

The seed coat (testa) of higher plants protects the embryo against adverse environmental conditions including germination control through dormancy imposition and by limiting the detrimental activity of physical and biological agents during seed storage. We subsequently speculated that there may be significant differences between WT and *pscb* mutant in seed water uptake and germination. To test this hypothesis, the water uptake and germination experiments were carried out in the WT and the *pscb* mutant. After keeping seeds immersed in water for 0.5 h, the *pscb* mutant absorbed 11.19% of the seed dry weight water while it was only 7.87% in wild type. The water absorption in *pscb* mutant was recorded 14.75, 17.69, 21.16, 24.37, 27.75, 38.94, 42.32, 44.06, 54.12, 54.12, and 54.12% at time interval of 1, 1.5, 2, 2.5, 3.5, 4.5, 5.5, 6.5, 7.5, 18.5, 19.5, and 20.5 h, respectively. While at each paired time points, the water absorbed by the wild type was 1.58–6.43% lower than that in *pscb* mutant (Figure 2A). After absorption of enough water, the seeds of *pscb* mutant and wild type were transferred to a 25°C incubator. The germination rate of wild type was 29.40% as compared to only 15.4% observed in *pscb* mutant, the differences enlarged till 24 h and 36 h and then narrowed at 48 h. Till 60 h, all the seeds germinated (Figure 2B) and only the length of bacon differed between wild type and pscb mutant (Figure 2C), In other words, the results showed that the *pscb* mutant had faster water uptake efficiency and delayed germination compared to WT.

### Estimation of lignin, anthocyanin, and proanthocyanidin contents

**Figure 3:**
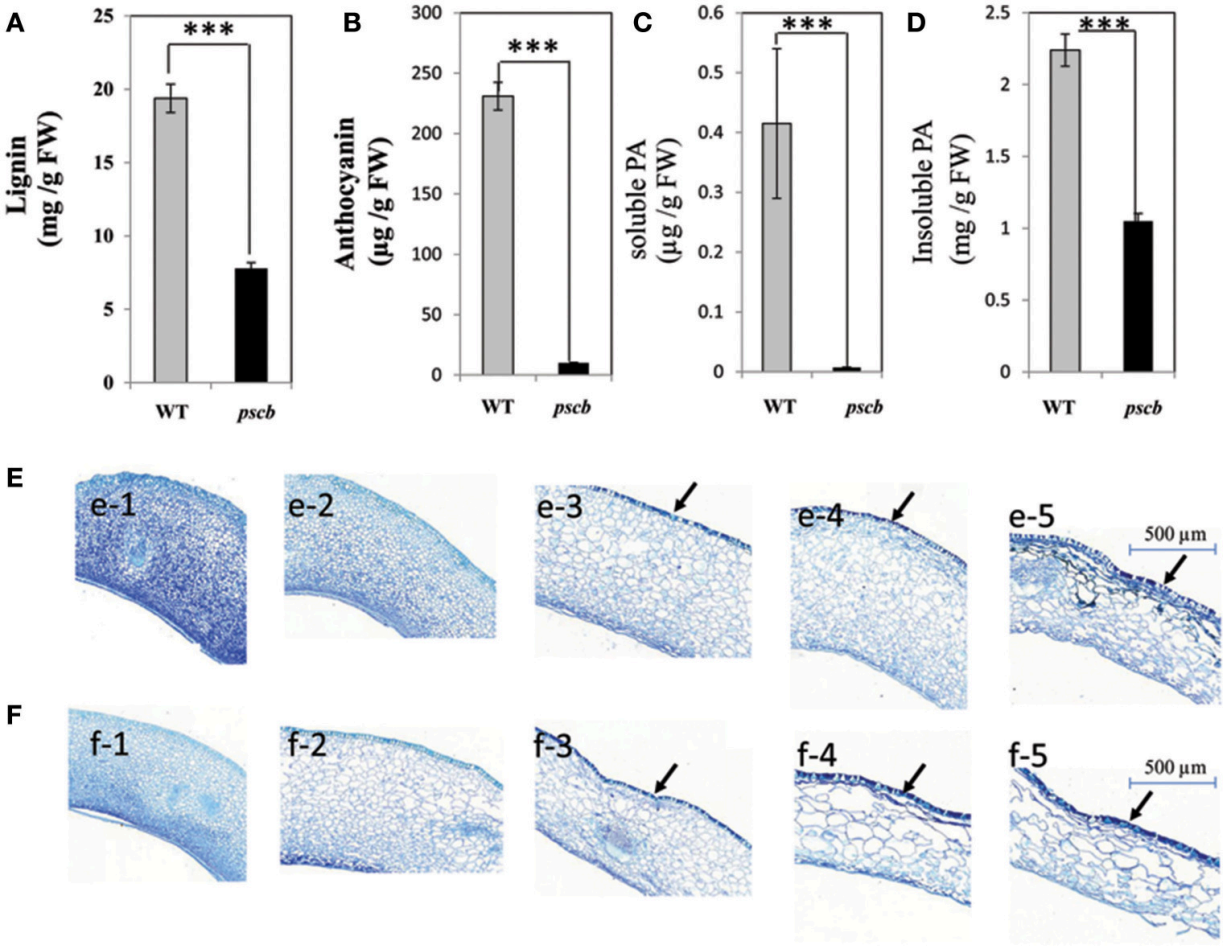
**The ***pscb*** mutant has decreased lignin, anthocyanin, and Proanthocyanidin content. (A)** Lignin content of matured seed coat in *pscb* mutant and WT. **(B)** Anthocyanin levels from *pscb* mutant and WT matured seed coat. **(C)** Soluble PA levels of *pscb* mutant and WT seed coat. **(D)** In-soluble PA content from *pscb* mutant and WT matured seed coat. FW, Fresh weight. **(E,F)** Changes of proanthocyanidins and phenolic compounds during seed coat development of *pscb* mutant and WT. **(e1–e5)** Detection and localization of proanthocyanidin and phenolic compounds in seed coat development in *pscb* mutant. Bars = 500 μm. **(f1–f5)** Detection and localization of proanthocyanidin and phenolic compounds in seed coat development in WT. Bars = 500 μm. Black arrows, accumulation site for polymeric phenolic compounds. ^***^Means significant differences.

It is well-known fact that the seed crack is related to physiological and ultimate structure of the cell wall. Since the lignin in the early stages of peanut seed coat is extremely low in quantity to detect, we analyzed the lignin content of the mature seed coat of the *pscb* mutant and the WT. The results showed that the lignin content in *pscb* mutant was 40.2% less as compared to WT (from 19.38 to 7.79 mg/g FW; Figure 3A). The *pscb* mutant showed brown seed coat color from DAF40, indicating change in the seed coat pigments. Direct measurement of anthocyanins and PAs content confirmed significant difference between *pscb* mutant and WT seed coat extracts (Figures 3B,D). The anthocyanins content was obviously lower i.e., 9.9 μg/g FW in *pscb* mutant as compared to WT i.e., 231 μg/g FW (Figure 3B). To quantify PAs accumulation, we extracted soluble and insoluble (non-extractable) PAs separately. Soluble PAs content in WT was 0.415 μg/g FW whereas the level of soluble PAs in *pscb* mutant was almost undetectable i.e., mere 0.0076 μg/g FW (Figure 3C). Measurement of insoluble PAs based on butanol-HCl hydrolysis showed that *pscb* mutant had less non-extractable PAs (Figure 3D).

**Figure 4:**
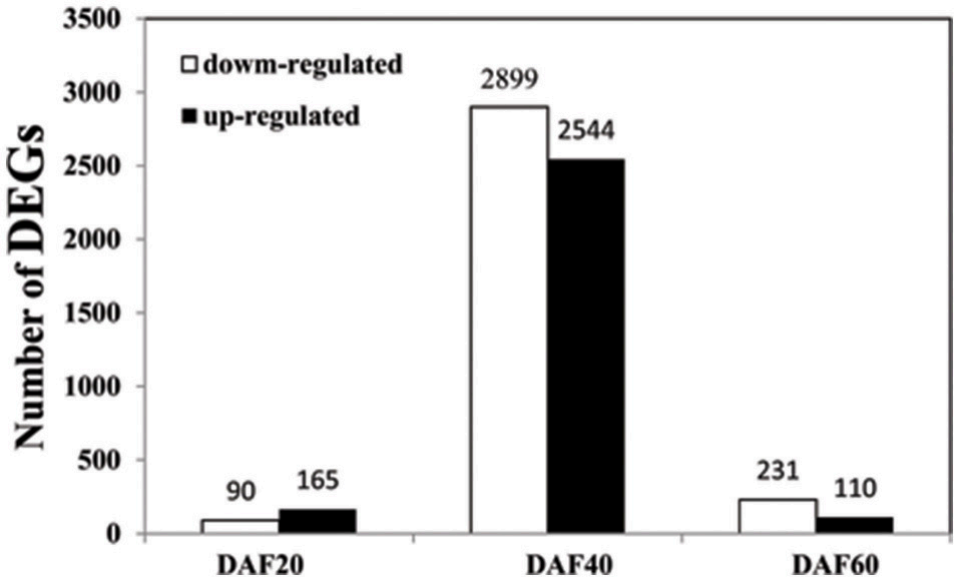
**Number of DEGs between ***pscb*** mutant and WT at DAF20, DAF40, and DAF60**.

**Figure 5:**
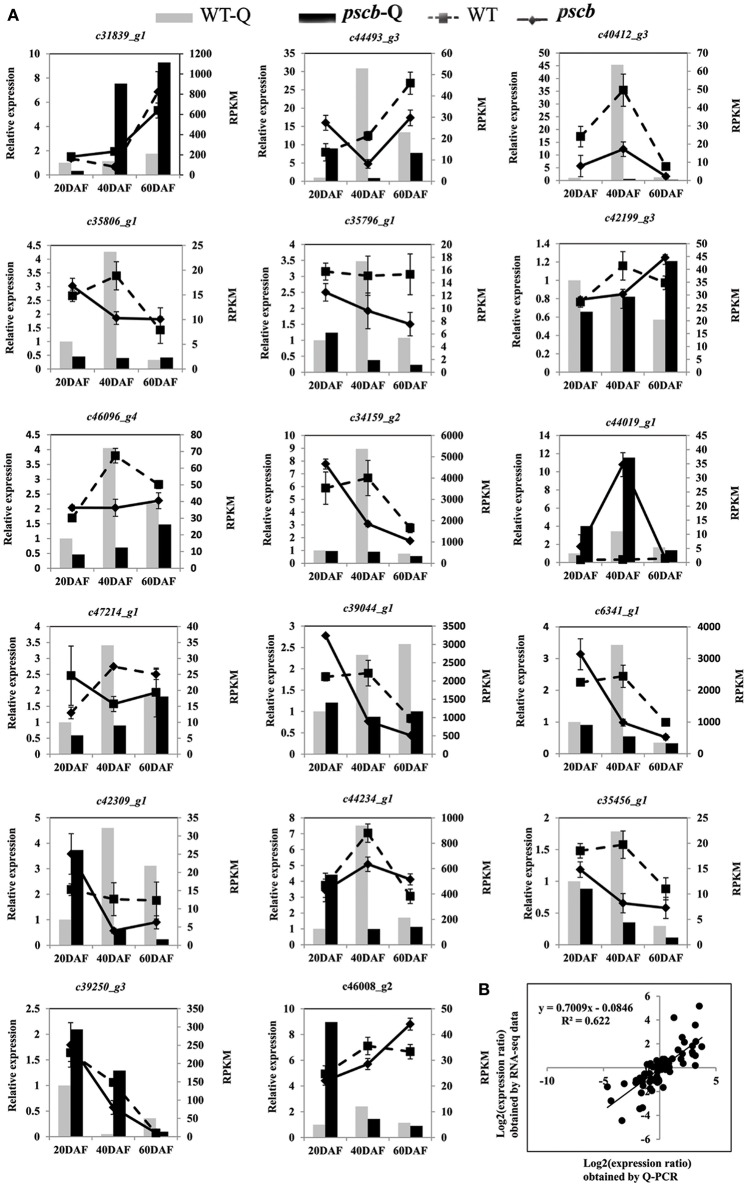
**qRT-PCR validation of differential expression. (A)** Transcript levels of 17 genes, which were involved in plant cell wall organization **(B)** Comparison between the gene expression ratios obtained from RNA-seq data and qRT-PCR. The RNA-seq log_2_ value of the expression ratio (*y*-axis) has been plotted against the developmental stages (*x*-axis).

To determine the cellular distribution of polyphenol compounds in developing seeds of *pscb* mutant and WT seed coat, a histochemical analysis was performed in the seed coat harvested at different developmental stages. TBO (toluidine blue O) staining of transverse sections of developing seeds revealed that at early seed development stages (DAF20 and DAF30), staining was similar in *pscb* mutant and WT (Figures 3e-1, e-2, f-1, f-2). At 40DAF, WT stained more intensely than the *pscb* mutant in the outer layer of the testa, indicating that more polymeric phenolic compounds had accumulated in WT (Figures 3e-3, f-3). The distribution of polymeric phenolic compounds was significantly different at the two late seed development stages (Figures 3e-4, e-5, f-4, f-5).

### Seed coat transcriptome differences between *pscb* mutant and WT during seed coat development

In order to understand the mechanism of seed coat development, we selected RNA samples of seed coat from three seed developmental stages i.e., DAF20, DAF40, and DAF60 showing different seed coat phenotype to perform the transcriptome analysis. The sequence data was deposited in the BioProject database of National Center for Biotechnology Information under the accession number PRJNA324725. One of the primary goals of transcriptome sequencing was to compare the gene expression levels in *pscb* mutant and WT. In this study, we used a stringent value of FDR ≤0.001 and fold change ≥2 as the threshold to judge the significant differences in the gene expression. A total of 5726 genes were found differently expressed between *pscb* mutant and WT (Table S1). Of these, 255 genes expressed at DAF20, 5443 genes at DAF40, and 341 genes at DAF60 (Figure 4). At DAF20, the number of up-regulated genes was more than the down-regulated genes (165:90). At DAF40, there was not much difference in the number of up-regulated and the down-regulated genes (2544:2899). At DAF60, the number of up-regulated genes was half of the number of the down-regulated genes (110:231). It was important to note that the number of DEGs increased significantly at the stage of DAF40, which was the vigorous growth stage during seed development (Figure 4).

To gain insights into the functional categories that were altered between *pscb* and WT (Zhonghua16), GO categories were assigned to the DEGs. Further, GO enrichment analysis of the DEGs in different developmental stages between the *pscb* mutant and WT (Zhonghua16) was performed for different developmental stages. Interestingly, no GO terms were enriched at DAF20, however, several significantly enriched terms in the biological process, molecular function, and cellular component categories were identified at DAF40 and DAF60 (Table 1). ADP binding (GO: 0043531), structural constituent of cell wall (GO: 0005199), plant-type cell wall organization or biogenesis (GO: 0071669), plant-type cell wall organization (GO: 0009664), and external encapsulating structure organization (GO: 0045229) were dominant terms at DAF60 in comparisons to DAF40. At DAF40, seed coat gets brown phenotype in the *pscb* mutant, indicating the difference between cell wall organizations might play an important role in the phenotype differentiation. At the DAF60, the main GO terms were related to fatty acid synthase and oxidoreductase activity (Table 1), including 3-oxoacyl-[acyl-carrier-protein] synthase activity (GO: 0004315), fatty acid synthase activity (GO: 0004312), fatty acid synthase complex (GO: 0005835), transferase activity, transferring acyl groups other than amino-acyl groups (GO: 0016747) etc. KEGG pathway analysis assigned the differential genes to 37, 287, and 46 metabolic pathways in three different developmental stages of *pscb* and Zhonghua 16. The complete list of metabolic pathways is provided in Table S2. Table 2 lists the metabolic/biological pathways in common of *pscb* compared with WT. Notably, among the 16 common KEGGs, 6 were involved in Tyrosine, Tryptophan, and Phenylalanine metabolism (Table 2).

**Table 1:** **Functional categorization of genes with significant transcriptional differences between ***pscb*** mutant and WT**.

	**GO accession**	**Description**	Term type^*^	**Corrected *p-*value**	**DEG item**
DAF40	GO:0043531	ADP binding	MF	8.55E-08	100
	GO:0005199	Structural constituent of cell wall	MF	0.00025097	15
	GO:0071669	Plant-type cell wall organization or biogenesis	BP	0.0061632	18
	GO:0009664	Plant-type cell wall organization	BP	0.013663	17
	GO:0045229	External encapsulating structure organization	BP	0.031101	30
	GO:0071555	Cell wall organization	BP	0.041254	28
	GO:0030312	External encapsulating structure	CC	0.041254	49
DAF60	GO:0004315	3-oxoacyl-[acyl-carrier-protein] synthase activity	MF	0.0066424	5
	GO:0004312	Fatty acid synthase activity	MF	0.0066424	5
	GO:0016747	Transferase activity, transferring acyl groups other than amino-acyl groups	MF	0.012673	12
	GO:0016706	Oxidoreductase activity, acting on paired donors, with incorporation or reduction of molecular oxygen, 2-oxoglutarate as one donor, and incorporation of one atom each of oxygen into both donors	MF	0.012673	8
	GO:0016491	Oxidoreductase activity	MF	0.012673	40
	GO:0016705	Oxidoreductase activity, acting on paired donors, with incorporation, or reduction of molecular oxygen	MF	0.016231	15
	GO:0043115	Precorrin-2 dehydrogenase activity	MF	0.016392	5
	GO:0003824	Catalytic activity	MF	0.020356	127
	GO:0008171	O-methyltransferase activity	MF	0.020356	6
	GO:0016628	Oxidoreductase activity, acting on the CH-CH group of donors, NAD or NADP as acceptor	MF	0.020356	6
	GO:0006633	Fatty acid biosynthetic process	BP	0.012673	10
	GO:0072330	Monocarboxylic acid biosynthetic process	BP	0.016392	10
	GO:0019354	Siroheme biosynthetic process	BP	0.016392	5
	GO:0046156	Siroheme metabolic process	BP	0.016392	5
	GO:0008610	Lipid biosynthetic process	BP	0.017694	19
	GO:0006631	Fatty acid metabolic process	BP	0.020356	10
	GO:0044710	Single-organism metabolic process	BP	0.020356	73
	GO:0055114	Oxidation-reduction process	BP	0.028844	38
	GO:0006629	Lipid metabolic process	BP	0.030943	26
	GO:0005835	Fatty acid synthase complex	CC	0.0066424	5

The genes were categorized based on GO.

* MF, molecular function; BP, biological process; CC, cellular component.

**Table 2:** **KEGG pathways in common of ***pscb*** mutant compared with WT at DAF20, DAF40, and DAF60**.

**KEGG pathway**	**Gene number**		
	**DAF20**	**DAF40**	**DAF60**
**Fatty acid elongation**	**1**	**6**	**7**
Isoquinoline alkaloid biosynthesis	1	2	1
Tropane, piperidine and pyridine alkaloid biosynthesis	1	2	1
Stilbenoid, diarylheptanoid and gingerol biosynthesis	1	5	1
**Tyrosine metabolism**	1	8	1
Beta-Alanine metabolism	1	10	1
Cyanoamino acid metabolism	1	10	1
Tryptophan metabolism	1	11	1
**Flavonoid biosynthesis**	**3**	**11**	**9**
Amino sugar and nucleotide sugar metabolism	3	13	1
**Phenylalanine metabolism**	**2**	**20**	**6**
Pyrimidine metabolism	1	31	1
**Phenylpropanoid biosynthesis**	**2**	**33**	**6**
Oxidative phosphorylation	1	38	1
Protein processing in endoplasmic reticulum	1	50	2
**Plant hormone signal transduction**	**1**	**56**	**7**

Bold means important KEGGs related to the phenotype.

### Verification of differentially expressed genes during seed coat development in *pscb* mutant and WT

Transcriptional regulation revealed by RNA-seq data was confirmed in a biologically independent experiment using the quantitative reverse transcription PCR. A total of 17 genes related to cell wall organization were selected to design gene-specific primers (Table S3) for real time PCR analysis (Figure 5). A linear regression analysis showed an overall correlation coefficient of *R* = 0.622, which indicated a good correlation between transcript abundance assayed by real-time PCR and the transcription profile revealed by RNA-seq data (Figure 5B).

### The *pscb* mutant seed coat accumulates phytomelanin through higher level of polyphenol oxidases and peroxidase expression

Various methods were used to solubilize and characterize the molecule(s) imparting the seed coat color that contribute to the brown pigmentation in *pscb* mutant seed. Compounds comprised of anthocyanins and proanthocyanidin were eliminated as candidates since their contents in *pscb* mutant were much lower than WT. Both bleach and peroxide were capable of removing the brown testa color of the *pscb* mutant seed coat. The intransigent nature of the dark compound, particularly its stability under acid hydrolysis and its susceptibility to the two treatments mentioned above, were reported as hallmarks of melanin, a class of chemically resistant phenolic polymers.

**Figure 6:**
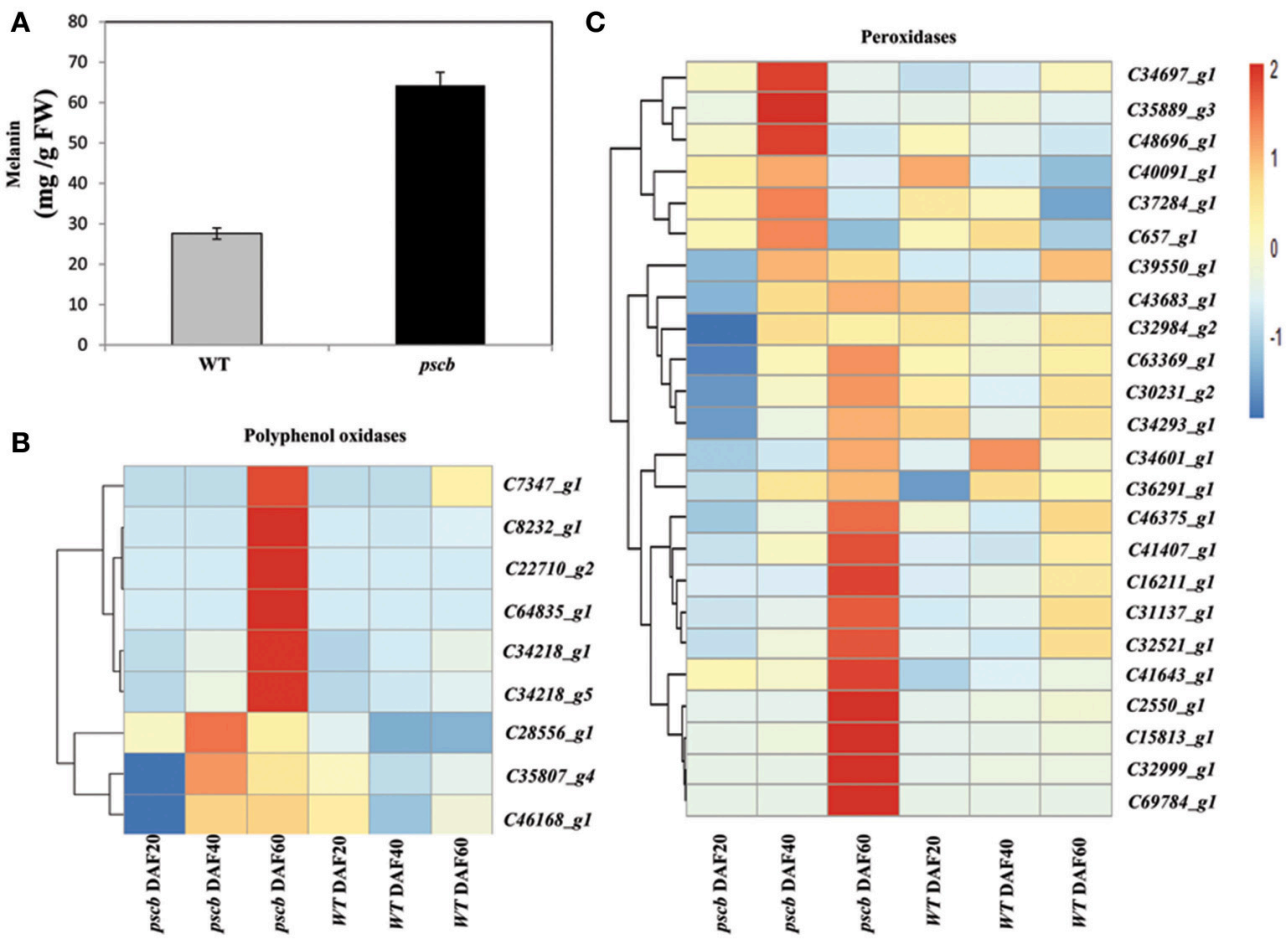
**The ***pscb*** mutant testa accumulates phytomelanin through higher level of peroxidase and polyphenol oxidase expression. (A)** Phytomelanin contents in *pscb* mutant and WT matured seed coat. The phytomelanin content was expressed as the mg g-1 fresh weight of seed coat. **(B)** Heatmaps represent the expression level of nine peroxidases in *pscb* mutant and WT seed coat of DAF20, DAF40, and DAF60. **(C)** Heatmaps represent the expression level of 24 polyphenol oxidase in *pscb* mutant and WT seed coat of DAF20, DAF40, and DAF60.The gene expression was scaled using Z-score of FPKM in the heatmap.

WT and *pscb* seed coat, when hydrolyzed with NaOH, produced little or no precipitate when the hydrolysates were subsequently acidified to pH 2. Contrasted with hydrolysates, both the *pscb* and WT seed coat produced abundant precipitates upon acidification. Furthermore, the precipitates could be resolubilized in NaOH or in dimethyl sulfoxide (DMSO), consistent with the hypothesis that the black pigment was melanic in nature, means both the WT and *pscb* seed coat had melanin constituents. The melanin content in *pscb* was 64.3 mg/g FW as compared to WT i.e., 27.6 mg/g FW (Figure 6A). Among the DEGs of three different stages of *pscb* and WT, there were nine polyphenol oxidases (PPOD) (Figure 6B, Table S4) and 24 peroxidases (POD) (Figure 6C, Figure S2, Table S4) obviously increased during the late developmental stages especially at DAF60 when compared with the WT. Most of the PPOD and POD showed different expression patterns among the developing process between *pscb* mutant and WT. In the *pscb* mutant, these genes had higher expression level during the seed coat development, while these genes either declined or remained stable in WT.

### Transcriptional regulation of ABA and ethylene signal transduction related genes during peanut seed coat development

The KEGG analysis of the RNA-seq data indicated significant change in the expression of ABA and ethylene signal transduction related genes in peanut seed coat, especially the genes of ABA signal transduction pathways. Previous study showed that ABA and ethylene in the maturation process play important roles, and between them there was a very close interaction. To better understand the transcriptional regulation of ABA and ethylene-related genes in peanut seed coat development, genes related to ABA and ethylene signal transduction were analyzed in the three different developing stages of WT and *pscb* mutant seed coat. At the early developing stage (DAF20), there was no DEG related to ABA and ethylene signal transduction. However, at DAF40, there were 13 DEGs in the ABA signal transduction between the *pscb* mutant and WT, including seven PYR/PYL family abscisic acid receptors (down-regulated), five PP2C (three down-regulated and two up-regulated), and two SnRK2 proteins (one up-regulated and one down regulated) (Table S5), showing that the ABA signal transduction was weakened in the mutant. The analysis of ethylene transduction related genes revealed a differential regulation between *pscb* mutant and wild type. The overall expression patterns of the genes in ethylene signal transduction were almost the same which were increased or kept invariant in WT and down-regulated in *pscb* mutant (Table S5). Such as ethylene-responsive transcription factor 1 (c31399_g2), the expression level stabilized around 2 (2.68, 1.96, 2.51) FPRK in WT of three stages, while in *pscb* mutant, declined from 1.32 to 0.12 FPRK.

### Candidate genes related to peanut crack and brown seed coat in *pscb*

In order to identify candidate genes controlling seed-coat cracking and seed color, we analyzed common DEGs between WT and *pscb* mutant from the three different developmental stages. The above analysis resulted in identification of 62 unigenes in WT and *pscb* mutant (Figure S1, Table S6). Among the common DEGs, we found three putative candidate genes (*c36498_g1:CCoAOMT1, c40902_g2:kinesin*, and *c33560_g1:MYB3*), which were significantly down-regulated in the *pscb* (Table S6). In the seed coat of wild type, the FPRK value of *c36498_g1* gene decreased from 10.96 (DAF20) to 3.38 (DAF40) and then to 8.59. In case of the *pscb* mutant, the FPRK value of *c36498_g1* gene declined from 0.58 (DAF20) to 0.24 (DAF40) and then to 0.03 (DAF60). The expression of *c40902_g2* gene in WT went up gradually from 5.31 (DAF20) to 7.29 (DAF40) and then 18.04 (DAF60). In contrast, the expression level of *c40902_g2* in *pscb* mutant went down from 0.38 (DAF20) to 0.34 (DAF40) and then to 0.17 (DAF60). Very few reads of *c33560_g1* gene were detected in *pscb* mutant, the FPRK were 0.08 (DAF20), 0.07 (DAF40), and 0 (DAF60), while in case of wild type, the FPRK value changed from 5.00 (DAF20) to 2.67 (DAF40) and then 2.77 (DAF60). C36498_g1 was a caffeoyl-CoA O-methyltransferase which was reported in Phenylalanine metabolism. The *CCoAOMT1* gene from maize, medicago, jute, poplar, *Zinnia elegans, Arabidopsis*, and loblolly pine were involved in lignin biosynthesis and cell wall organization (Ye et al., 1994, 2001; Ye and Varner, 1995; Goujon et al., 2003; Zhou et al., 2010; Wagner et al., 2011; Li et al., 2013; Zhang et al., 2014). *C40902_g2* encode a kinesin-4-like protein, and kinesin protein were reported functioned in cell wall organization (Zhong et al., 2002; Zhang et al., 2010; Fujikura et al., 2014; Kong et al., 2015). *c33560_g1* was an R2R3-Myb transcription factor encoding gene. Previous studies showed R2R3-Myb factor worked combined with other transcription factors together to regulate flavonoids and phenylalanine metabolism further regulate the proanthocyanidin biosynthesis (Baudry et al., 2006; Quattrocchio et al., 2006). These genes that might lead to the brown seed color and crack seed coat phenotype need to be confirmed in further functional genomics studies.

## Discussion

The conventional understanding of the role of the seed coat is that it provides a protective layer for the developing zygote. It also acts as channel for transmitting environmental cues to the interior of the seed which helps seed to adjust its metabolism in response to changes in its external environment (Radchuk and Borisjuk, 2014). In peanut, flavonoid, and phenylpropanoid biosynthesis pathways were reported to be related with aflatoxin resistance (Garcia et al., 2013; Wang et al., 2016). Therefore, the research on seed coat cracking and pigmentation/color will not only help in understanding and improving the peanut seed quality, it will also help in understanding the genetic control for few seed borne diseases such as aflatoxin contamination. In the present study, RNA-seq was used to investigate the differences in the transcriptome between the three different stages of *pscb* mutant and its WT. Thousands of genes that were differently regulated during the three stages of seed coat development were identified by transcriptomic profiling.

### Seed coat pigmentation was redirected in *pscb* mutant between anthocyanin, proanthocyanidin, melanin, and lignin

Flavonoids are the secondary metabolites that accumulate in plants and promote seed and pollen dispersal by contributing to color formation in fruits and flowers (Winkel-Shirley, 2001). Previously, researchers showed that epicatechin derivatives (Marinova et al., 2007; Zhao and Dixon, 2009) and a PA monomer (Holton and Cornish, 1995; Grotewold, 2006) are important flavonols in the synthesis of the seed coat of *Arabidopsis* and *Medicago*. Originally when we first observed the *pscb* mutant, we thought the pigmentation must be markedly enhanced when compared with WT for the sight of deeper color. However, compounds comprised of anthocyanins and lignins were eliminated as candidates, in contrast the production of anthocyanins and proanthocyanidins was reduced in the *pscb* mutant seed coats. We noticed that both bleach and peroxide were capable of removing the dark testa color of the *pscb* mutant seed and the dark compound was stable under acid hydrolysis, these features were reported as hallmarks of melanin (Fogarty and Tobin, 1996; Sava et al., 2001), a class of chemically resistant phenolic polymers. Melanin, was reported as black pigments, especially in the seed coat of composite, morning glory and many oilseed *Brassica* species (Park et al., 2007; Park and Hoshino, 2012; Yu, 2013). Surprisingly, the melanin content in the *pscb* mutant was more than twice as compared to WT. The seed coat crack and brown color might be contributing for the inhibition of flavonoid and lignin metabolism pathway, and the accumulation of the upper component of the aromatic amino acid converted to be melanin and compensating for the disadvantages of the lower anthocyanin and proanthocyanidin content.

### Potential mechanisms underlying seed coat color and crack in peanut

Flavonoids are plant secondary metabolites derived from the phenylpropanoid pathway. The flavonoid pathway in *Arabidopsis* has been characterized mainly using the mutants. Twenty-three genes have already been identified at the molecular level corresponding to several enzymes (CHS, CHI, F3H, F3'H, DFR, LDOX, FLS, ANR, LACCASE), transports (TT12, TT19, AHA10), and regulatory factors (TT1, TT2, TT8, TT16, TTG1, TTG2, PAP1, GL3, ANL2, FUSCA3, KAN4) (Baxter et al., 2005; Li et al., 2010). The transcriptome data generated in this study showed that the *F3H, F3*′*H, DFR*, and *ANR* were suppressed in the stage of DAF40. These results suggested that DAF 40 was the key growth stage for the anthocyanins and proanthocyanidins accumulation. Further, *F3H, F3*′*H, DFR*, and *ANR* are the key genes underlying differences in seed coat pigmentation in *pscb* mutant and WT.

**Figure 7:**
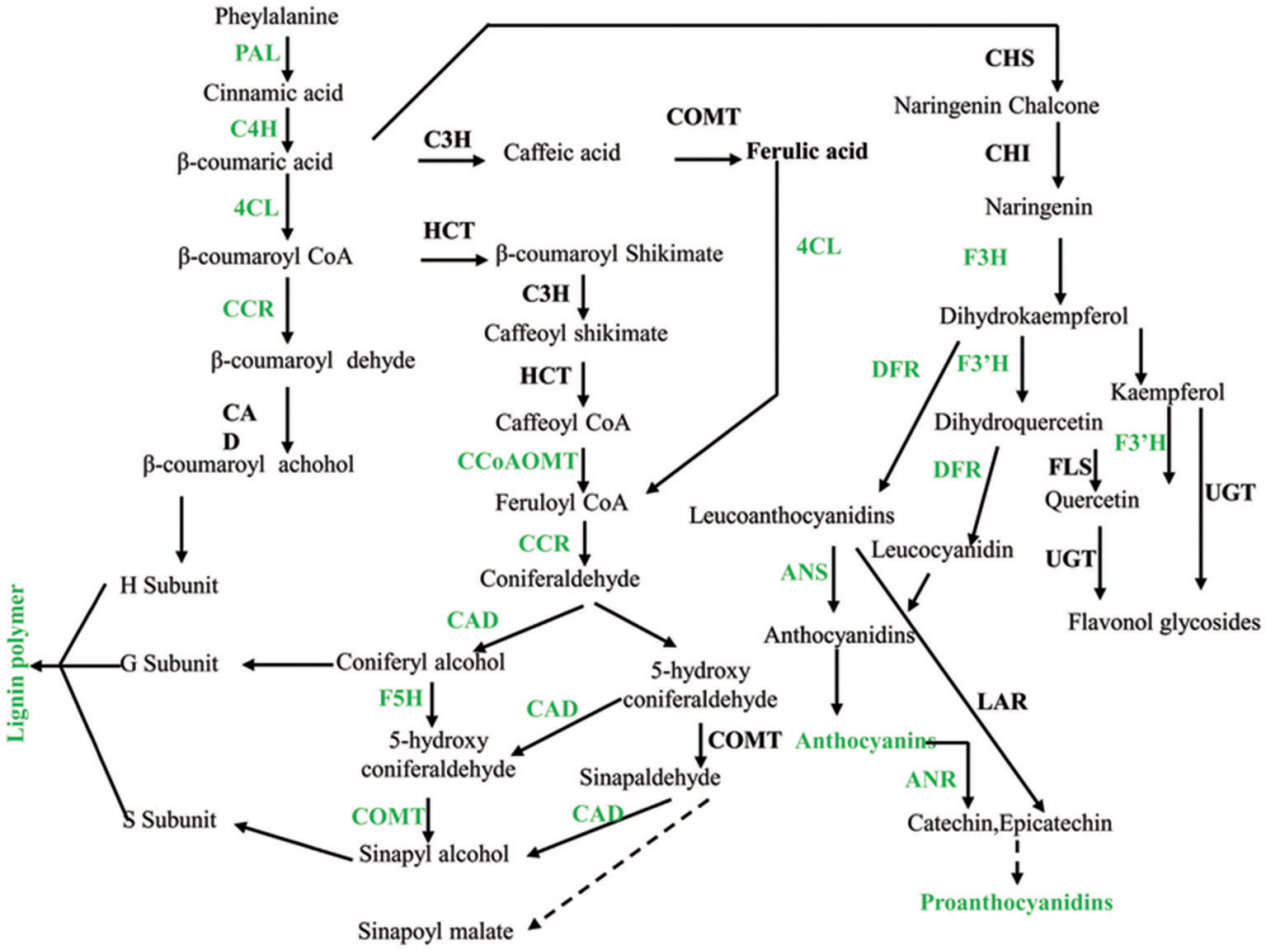
**Phenylalanine metabolism was down-regulated in ***pscb*** mutant seed coat**. PAL, phenylalanine ammonia-lyase; C4H, cinnamate 4-hydroxylase; 4CL, 4-coumarate-CoA ligase; CCR, cinnamoyl-CoA reductase; CAD, cinnamyl alcohol dehydrogenase; HCT, hydroxycinnamoyl-CoA shikimate/quinate hydroxycinnamoyl transferase; C3H, 4-coumarate 3-hydroxylase; COMT, caffeic acid o-methyltransferase; CCoAOMT, caffeoyl-CoA o-methyltransferase; F5H, ferulate-5-hydroxylase. CHS, chalcone synthase; CHI, chalcone isomerase; F3H, flavanone 3-hydroxylase; F3′H, flavonoid 3′-hydroxylase; FLS, flavonol synthase; UGTs, UDP sugar glycosyltransferases; DFR, dihydroflavonol reductase.

Melanin production resulting in black pigmentation proceeds by one of two pathways in plants. The first leads to compounds of the allomelanin variety deviating in the shikimic acid pathway at β-coumarate before the flavonoids (Goodwin and Mercer, 1983). The second produces eumelanin from Tyr via oxidation of DL-dioxy-Phe and is divorced from the shikimate pathway altogether. Polymerization of melanic compounds produces peroxide (Blois, 1978) and trapped free radicals that should lead to an up-regulation of PRX activity and an EPR signal, respectively. The transcriptome analysis revealed that many structural genes which are involved in the phenylalanine metabolism were found down-regulated in the *pscb* mutant (Figure 7) in addition to decrease of the lignin, anthocyanin, and proanthocyanidin content. In contrast, there were also nine polyphenol oxidases and 24 peroxidases obviously increased at the late developmental stages especially at DAF60 when compared with the WT.

It has already been reported that the down regulation of C3H, 4CL1, F5H, or COMT enzymes in *A. thaliana* affects the final lignin composition (Chapple et al., 1994; Lee et al., 1997; Meyer et al., 1998; Franke et al., 2002; Goujon et al., 2003). The repression of this set of genes in transgenic plant leads to a 70% reduction in the total lignin content and resulted in severe phenotypic effects. We recently described that the lignin content in the *pscb* mutant decreased by 60%, this strong reduction in lignin content can be related with the down regulation of genes involved in lignin synthesis pathway such as *CAD, F5H*, or *COMT.* The above results indicate that the synthesis mechanism of lignin in peanut is similar to other plants.

Previous study hold the idea that lignin is related to PA due to common steps in the phenylpropanoid pathway. The low lignin is found strongly associated with the unpigmented seed coat trait as lignin is usually accumulated within the cell wall whereas PA is usually accumulated in endoplasmic reticulum vesicles or in the plant vacuole. Lignin variability may influence seed coat pigment extractability, owing to the position of the highly lignified palisade cells adjacent to the inner integument in the seed coat, where pigment is initially deposited (Marles and Gruber, 2004). Here, our result showed that the anthocyanins, proanthocyanidins, and the lignin content were reduced while the melanin content was enhanced, indicating new regulation mechanism may exist in peanut seed coat.

### Regulation mechanisms underlying the biosynthesis of seed coat pigment in peanut

Plant hormone and transcription factors play an important role in plant seed development. Through the analysis of the RNA-seq data, we found that hormone-related genes in the *pscb* mutant seed coat during seed development changed greatly when compared with WT especially the ABA and ethylene signal transduction pathway. Plant hormone ABA has been suggested to play a role in fruit anthocyanin biosynthesis (Shen et al., 2014; Li et al., 2015). Our transcriptome data showed that all the seven ABA receptor PYR/PYL genes, which presented in the KEGG “plant hormone signal transduction,” were obviously down regulated in the *pscb* mutant indicating the ABA signal transduction might be strongly suppressed in the mutant leading to the lower anthocyanin and proanthocyanidin content. Ethylene is required for the onset of accumulation of anthocyanins (Chervin et al., 2004). In this study, one ETR1, three EBF1, two EIN3, and two ERF1 involved in ethylene signal transduction were down regulated at DAF40 in *pscb* mutant. Further, the three EBF1 and two ERF1 were also down-regulated in the stage of DAF60, which strongly demonstrate that the ethylene signaling was suppressed in the mutant. MYB transcription factors have been well-reported in the regulation of plant pigmentation in different species (Appelhagen et al., 2011; Liu et al., 2014; An et al., 2015; Cavallini et al., 2015; Yoshida et al., 2015). Through analysis of the common DEGs of the three different stages, we identified a R2R3-MYB transcription factor (c33560_g1), with extremely low expression in the stages of DAF20 and DAF40 and none expression in the late stage of DAF60 in *pscb* mutant. By blasting the protein sequence of c33560_g1 in The *Arabidopsis* Information Resource (TAIR), AT1G22640 (MYB3) showed the highest similarity, while AT1G22640 was reported as an MYB-type transcription factor (MYB3) that represses phenylpropanoid biosynthesis gene expression (Rowan et al., 2009). The MdMYB3, the homolog in apple, was similarly identified as regulator of anthocyanin biosynthesis and flower development (Vimolmangkang et al., 2013), indicating the potential function of *c33560_g1* in peanut seed coat pigmentation.

**Figure 8:**
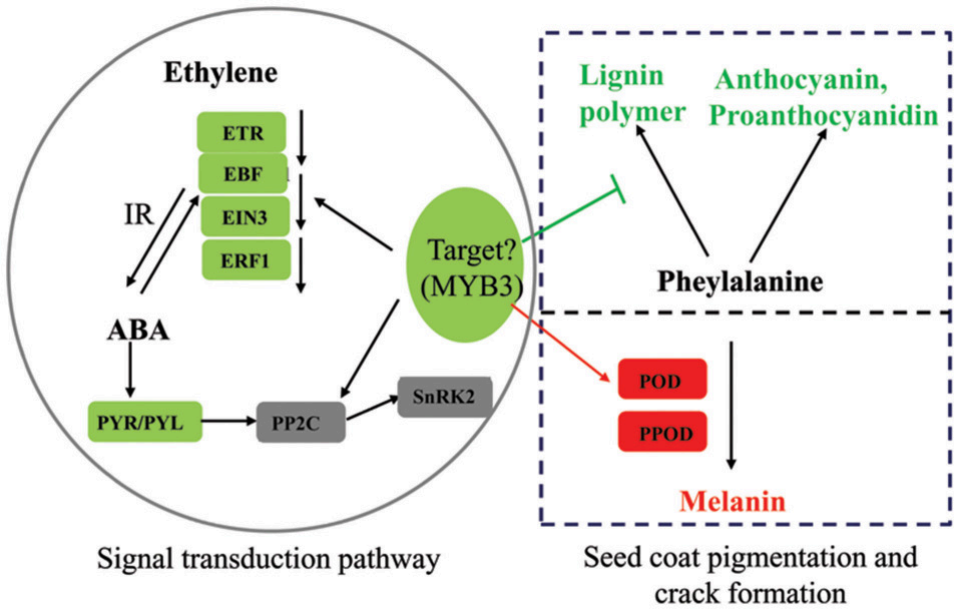
**Summary of some biological pathways involved in peanut seed coat pigmentation and crack formation**. Red boxes indicate genes/proteins that were upregulated in *pscb* mutant compared with WT; green boxes indicate genes/proteins that were downregulated in *pscb* mutant compared with WT, 1-aminocyclopropane-1-carboxylate oxidase; ETR, ethylene receptor; EBF, EIN3-binding F-box protein; ERF1, ethylene-responsive transcription factor; PYR/PYL, abscisic acid receptor; PP2C, protein phosphatase 2C; SnRK2, serine/threonine-protein kinase SRK2; POD, peroxidase; PPOD, polyphenol oxidase; IR, interaction.

In the present study, the ABA pathway, ethylene pathway, and the R2R3-MYB transcription factor (c33560_g1) were all different between *pscb* mutant and WT and were selected as important candidate for the format of the cracking and brown seed coat phenotype. We hypothesize that the R2R3-MYB transcription factor (c33560_g1), ABA and ethylene signaling pathways interact cooperatively to suppress the anthocyanin, proanthocyanidin, and lignin synthesis related pathways' genes to influence the anthocyanin, proanthocyanidin and lignin level. Simultaneously, the enhanced expression of POD and PPOD encoding genes further regulate seed coat pigmentation (Figure 8). The above does not prove the interaction relationship of the three factors responsible for seed-coat cracking and brown seed color trait of *pscb* mutant and requires further detailed study.

## Author contributions

Conceived and designed the experiments: LW, YL, LY, HJ, BSL. Performed the experiments: LW, YW, BL. Analyzed the data: LW, MP. Contributed reagents/materials/analysis tools: GW, XD, YL, LY, HJ. Wrote the paper: LW, MP, RV, BSL. All authors have read and approved the manuscript.

### Conflict of interest statement

The authors declare that the research was conducted in the absence of any commercial or financial relationships that could be construed as a potential conflict of interest.

## Supplementary material

Figure S1 **Venn diagram showing the number of genes with increased and decreased expression of three different development stages**.

Figure S2 **qPCR verification of the POD AND PPOD change in ***pscb*** mutant compared with WT**.

Table S1 **DEGs between ***pscb*** and WT at DAF20, DAF40, and DAF60**.

Table S2 **KEGG pathway of the DEGs between ***pscb*** mutant and WT at DAF20, DAF40, and DAF60**.

Table S3 **Gene-specific primers for real time PCR analysis**.

Table S4 **Expression and annotation the differential expressed POD and PPOD genes**.

Table S5 **Differentially expressed genes involved in ABA and ethylene signal transduction between ***pscb*** mutant and WT**.

Table S6 **Common DEGs between WT and ***pscb*** mutant of the three different developmental stages**.
