# Supplementary figures and images for: Transcriptome Analysis of a New Peanut Seed Coat Mutant for the Physiological Regulatory Mechanism Involved in Seed Coat Cracking and Pigmentation
Source: Front Plant Sci. 2016 Oct 14;7:1491. doi: 10.3389/fpls.2016.01491 (PMC5063860; doi:10.3389/fpls.2016.01491)

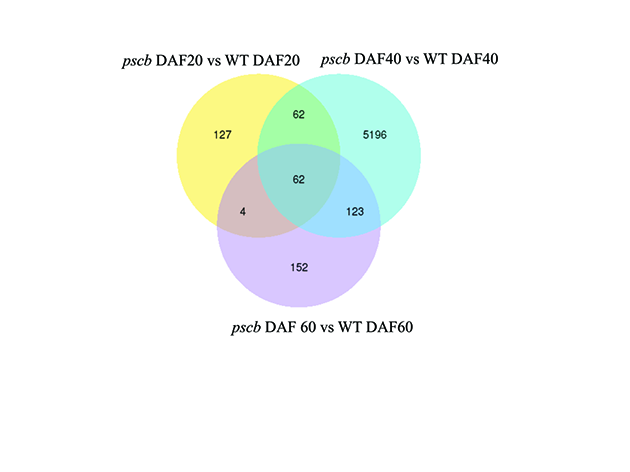

Supplement: Figure S1 — Venn diagram showing the number of genes with increased and decreased expression of three different development stages. [file Image1.TIF]

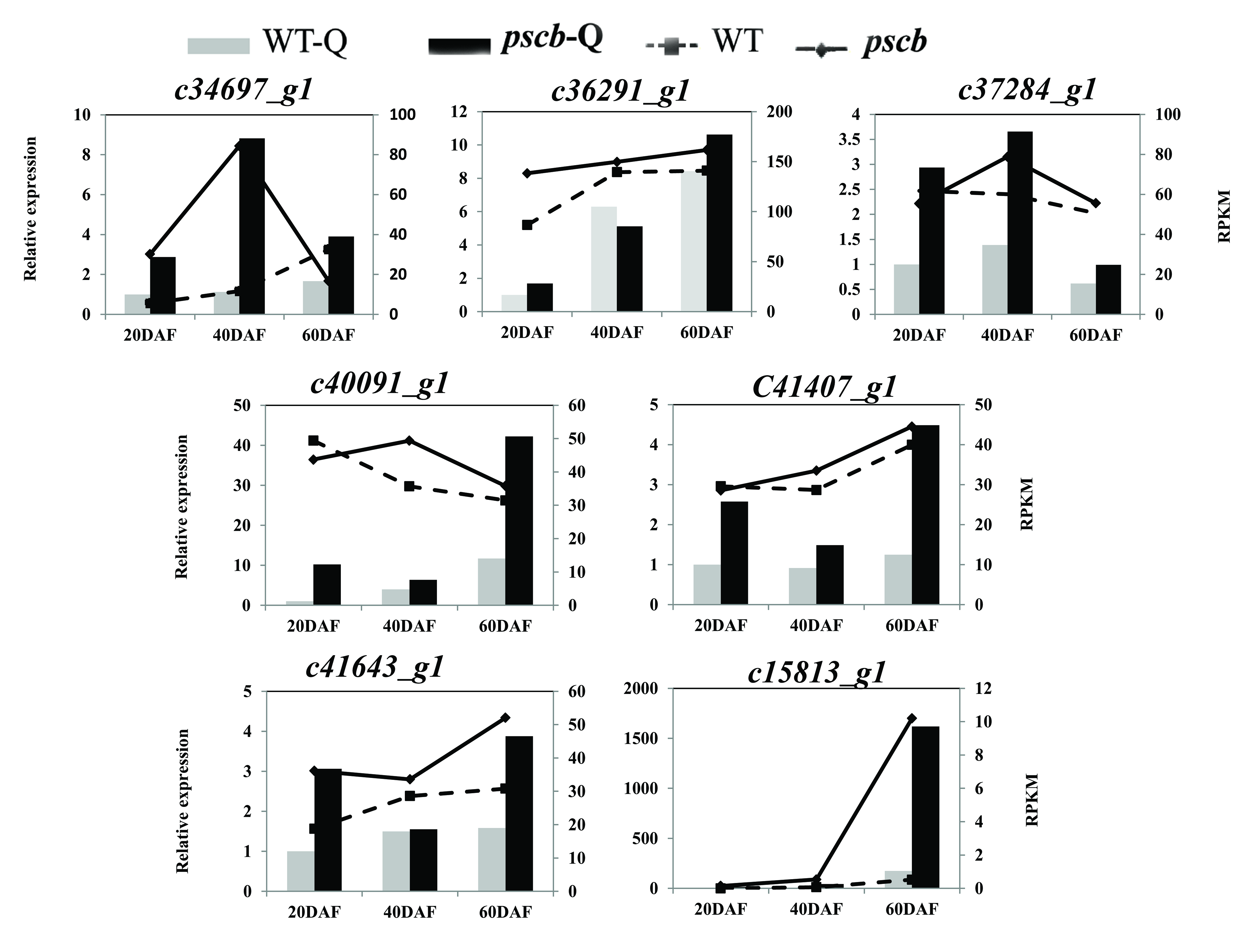

Supplement: Figure S2 — qPCR verification of the POD AND PPOD change in pscb mutant compared with WT. [file Image2.TIF]
